# Risk factor analysis in self-reported diabetes in a rural Kerala population

**DOI:** 10.4103/0973-3930.44080

**Published:** 2008

**Authors:** Rajnarayan R. Tiwari, Pankaj K. Deb, Aghore Debbarma, Rupali Chaudhuri, Amita Chakraborti, Mickyla Lepcha, Gita Chakraborti

**Affiliations:** National Institute of Occupational Health, Ahmedabad, India; 1 Govind Ballabh Pant Hospital, Agartala, India; 2 Tripura Health Services, Agartala, India; 3 Indira Gandhi Memorial Hospital, Agartala, India; 4 Sir Thutop Namgyal Memorial Hospital, Gangtok, India

**Keywords:** Kerala, self-reported diabetes mellitus, waist circumference

## Abstract

**AIM:**

To find the prevalence of self-reported diabetes mellitus (DM) in rural Kerala.

**METHODOLOGY:**

Interview schedule was used to collect the information on pre-designed and pre-tested proforma. Self-reported DM was taken as outcome measure. All these patients were diagnosed by their respective physicians and were on anti-diabetic drugs. Body weight was measured to the nearest 1 kg using bathroom scale; while height was measured using a nonstretchable tape to the nearest 1 mm. Standard classification of obesity and waist circumference was used.

**RESULTS:**

The present cross-sectional study was carried out in the Venganoor village of Kerala. Four hundred and sixty four subjects, 64.4% women and 35.6% men, were selected randomly. Nearly half of the subjects were in the age range of 25–54 years, while about one-third of the subjects were over 55 years. Among the men, 38.5% were ever smokers, while 5.5% were in the habit of consuming alcohol. 27.8% of the subjects were found to be obese, while 20.3% of the subjects reported raised waist circumference. The level of physical activity in the majority of the subjects was either sedentary or mild. On multivariate analysis, increasing age was found to be significantly associated with self-reported diabetes (OR = 1.07; 95% CI: 1.04–1.09). The other factors namely sex, smoking habit, alcohol use, obesity, waist circumference and physical activity were found to be statistically nonsignificant.

**CONCLUSION:**

The prevalence of self-reported diabetes was found to be 13.1% and that it was seen to be associated with increasing age.

## Introduction

Diabetes, a global public health problem, is now emerging as a pandemic. By the year 2025, three-quarters of the world's 300 million adults with diabetes will be in nonindustrialized countries and almost one third in India and China alone.[1] India has nearly 33 million patients suffering from diabetes today, which is briefly contributed by the urban population.[1] The scenario is changing rapidly due to socioeconomic transition also occurring in the rural areas.

Environmental and lifestyle changes resulting from industrialization and migration to urban environment from rural settings may be responsible for the increasing incidence of diabetes to a large extent. Availability of improved modes of transport has resulted in decreased physical activities. Better economic conditions have produced changes in dietary habits. The conditions are more favorable for expression of diabetes in the population, which already has a racial and genetic susceptibility to the disease. Recent epidemiological data show that the situations are similar throughout the country.[2]

The conversion to diabetes is enhanced by the low threshold for the risk factors, such as age, body mass index (BMI) and upper body adiposity. Indians have a genetic phenotype characterized by low BMI, but with high upper body adiposity, high body fat percentage and high level of insulin resistance. With a high genetic predisposition and the high susceptibility to the environmental insults, the Indian population faces a high risk for diabetes and its associated complications. As several of the factors associated with diabetes are potentially modifiable, this epidemic of diabetes can be curbed if proper measures are taken to increase physical activity and reduce obesity rates.

Though several studies have been carried out among urban population where the prevalence is rising, very few studies have been carried out among the rural population where the epidemiological transition is comparatively slow. In a random multistage cross-sectional population survey in subjects aged 25 years and above among 7,746 (3,629 men; 4,117 women) subjects from rural area, it was found that the standardized prevalence rate for DM in the rural populations was 2.7%.[3] Similarly, in another study among 1,769 rural (894 men; 875 women) subjects, the prevalence of DM was 2.8% using the criteria of World Health Organization.[4] Thus, the present study was carried out to find the prevalence of self-reported DM in those aged ≥15 years and to study some associated epidemiological factors.

## Methodology

The present cross-sectional study was carried out in one of the anganwadi areas of Venganoor, a village in Thiruvananthapuram district of Kerala during December 2004 to March 2005. This anganwadi area was randomly selected by simple random sampling method. This randomization included preparation of the list of gram panchayats in Thiruvananthapuram district of Kerala. By lottery method, the village-Venganoor was selected. Thereafter, one of the anganwadi areas was selected from the previously prepared list of anganwadis in the Venganoor, village by lottery method.

This randomly selected study area had 249 houses, of which 222 houses were included in the present study. The remaining houses could not be covered because they were locked and nobody could be found in spite of repeated efforts. Of 953 people residing in the 222 houses included in the study, 755 subjects were aged ≥15 years. In the present study, 467 subjects were included; thereby, giving a total population coverage of 61.9%.

Interview schedule was used to collect the information on pre-designed and pre-tested proforma. The information recorded included the demographic and socioeconomic characteristics as well as the information on risk factors. Self-reported DM was taken as outcome measure. All these patients were diagnosed by their respective physicians and were on anti-diabetic drugs. For the present study, all those who had smoked at least one cigarette or bidi in the last one-month period were considered as current smokers, while those who had quit smoking since ≥1 year were considered as ex-smokers. For analysis, the current smokers and ex-smokers were categorized in the “ever smoker” group. Similarly, those who reported to have consumed alcohol at least once in last one-month period were considered as current alcohol users. This was followed by measurement of blood pressure, height, weight and waist circumference.

Body weight was measured (to the nearest 0.01 kg) with the subject standing still on the electronic weighing scale, feet about 15 cm apart and weight equally distributed on each leg. Subjects were instructed to wear minimum outerwear (as culturally appropriate) and no footwear while their weight was being measured. Height was measured using a nonstretchable tape (to the nearest 0.1 cm) with the subject in an erect position against a vertical surface and the head positioned so that the top of the external auditory meatus was in level with the inferior margin of the bony orbit. Body mass index was calculated by dividing the weight (in kilograms) with the square of height (in meters). Standard classification of obesity was used for the categorization.[5]

Waist circumference (to the nearest 0.1 cm) was measured using a tailor's tape at a point mid way between tip of iliac crest and last costal margin in the back and at umbilicus in the front. IDF standard cut offs of ≥88 cm and ≥90 cm were used for women and men, respectively.[6] Statistical analysis was carried out using Epi Info version 3.3. Multivariate analysis was carried out to calculate adjusted Odds ratios (ORs) and their 95% confidence intervals.

## Results

Table 1 describes the prevalence of different risk factors among study subjects. The present study included 64.4% women and 35.6% men. Less number of men are due to the fact that they being the working members of the family were out on their job at the time of survey. However, repeated efforts to contact them including visits to their houses on Sundays went futile. Nearly half of the subjects belonged to the age group of 25–54 years, while about one-third subjects were above 55 years of age. None of the women reported habits of smoking or alcohol consumption. Among the men, 38.5% were “ever smokers”, while 5.5% were in the habit of consuming alcohol. Using BMI cut-off level of 25 kg/m^2^, 27.8% subjects were found to be obese. The mean BMI for women and men was found to be 23.4 ± 4.2 kg/m^2^ and 22.2 ± 4.4 kg/m^2^ respectively. The difference was found to be statistically nonsignificant. Waist circumference measured according to standard criteria for men and women, which is considered as an index of central obesity, was raised in 11.4% of the subjects. The level of physical activity in the majority of the subjects was either sedentary or mild.

**Table 1:** The prevalence of different risk factors among study subjects

Age (in years)	Men n = 165	Women n = 299	Total n = 464
15–24	34 (20.6)	38 (12.7)	72 (15.5)
25–34	27 (16.4)	70 (23.4)	97 (20.9)
35–44	26 (15.8)	61 (20.4)	87 (18.8)
45–54	22 (13.3)	57 (19.1)	79 (17.0)
55–64	33 (20.0)	37 (12.4)	70 (15.1)
≥64	23 (13.9)	36 (12.0)	59 (12.7)
Risk factors			
Smoking history*	44 (38.5)	-	44 (38.5)
Ever smokers	121(61.5)	-	121(61.5)
Never Smokers			
Alcohol use*			
Present	55 (5.5)	-	55 (5.5)
Absent	110 (94.5)	-	110 (94.5)
Body mass index#			
Obese	29 (17.5)	100 (33.1)	129 (27.8)
Nonobese	136 (82.5)	199 (66.9)	335 (72.2)
Waist circumference#			
Normal	131(79.4)	211 (70.6)	342 (73.7)
Abnormal	34(20.6)	88 (29.4)	122 (26.3)
Physical activity			
Moderate and heavy activity	104 (63)	77 (25.5)	181 (39.1)
Sedentary and mild activity	61 (37)	222 (74.5)	283 (60.9)

Figures in parentheses are percentages

* Include only males, n = 464 as data for three subjects was missing

Table 2 describes the association of the risk factors with the self-reported DM on multivariate analysis. Increasing age was found to be significantly associated with self-reported diabetes (OR = 1.07; 95% CI: 1.04–1.09). The other factors namely sex, smoking, alcoholism, obesity, waist circumference and physical activity were found to be statistically nonsignificant.

**Table 2:** Multivariate analysis of risk factors for self-reported diabetes mellitus

Risk factors	Adjusted OR (95% CI)
Age (in years)	1.07 (1.04–1.09)#
Male Sex	0.79 (0.37–1.67)
Ever smokers	0.97 (0.32–2.93)
Alcohol use*	1.28 (0.46–3.57)
Higher body mass index	1.02 (0. 93–1.12)
Higher waist circumference	1.04 (0.99–1.08)
Physical activity	0.99 (0.5–1.94)

# Statistically significant

* Included only males

## Discussion

In the present study, the prevalence of self-reported DM in the rural population of Kerala was found to be 13.1%. This is the crude way of estimating the prevalence of DM against the standard way of estimating it by oral glucose tolerance test. However, as the main objective was to have some baseline data on which further research could be planned, the present study was undertaken. Though the prevalence cannot be compared with the prevalence found in other studies as the methodology is different, the prevalence in the present study is higher than that reported for the rural areas in other studies. In an Indian study,[7] among 44,523 individuals (age range: 15–64 years) inclusive of 15,239 from urban, 15,760 from peri-urban/slum and 13,524 from rural areas, the lowest prevalence of self-reported diabetes was recorded in rural (3.1%), followed by peri-urban/slum (3.2%) and the highest in urban areas (7.3%; OR for urban areas: 2.48, 95% CI: 2.21–2.79; *P* < 0.001). In another study carried out at Thiruvanathapuram district of Kerala,[8] the overall crude prevalence rate of type 2 diabetes was reported to be 5.9%. It was highest in the urban (12.4%), followed by midland (8.1%), highland (5.8%) and coastal (2.5%) regions. Similarly, another community based cross-sectional survey[9] reported prevalence of known DM as 9.0%.

Age was found to be associated with the risk of DM. Those aged 45 years or more were found to have higher risk as compared to those younger than 45 years. Indians develop diabetes at a very young age, at least 10–15 years earlier than the Caucasian population. The national urban diabetes survey in India showed that more than 50% of the diabetic cases had the onset below the age of 50 years.[1,10] With advancing age, lean body mass decreases and percent adiposity increases, but there may be little or no change in the total body weight. Aging is associated with sarcopenia, referred to as the universal and involuntary decline in skeletal muscle mass. This results in loss of muscle strength and contributes to eventual inability of the elderly to carry out tasks of daily living. The chief function of insulin is to facilitate glucose uptake by the muscles. A reduction in lean body mass means the eventual inability to dispose glucose. Reduced metabolically active lean tissue mass and physical activity levels in older people predispose them to obesity. This further increases insulin resistance.[11,12]

Sex, smoking and alcoholism were found to be nonsignificant factors. A recent report of WHO has also mentioned that consumption of an average of ≥40 g pure alcohol per day for women and ≥60 g for men is nonsignificantly associated with diabetes.[13–14] We also found similar results in the present study. However, partly it may be attributed to the small sample size of the alcohol users and secondly, though the females were using the homemade alcoholic beverages, they were reluctant to mention that during the study due to social stigma. As the number of diabetics was more in the female group, this underreporting might have resulted in the nonsignificance of this factor.

In the present study, the association of obesity with the occurrence of DM could not be established. This may be attributed to the fact that the occurrence of diabetes is more associated with abdominal obesity rather than the overall obesity status.[15] The finding in the present study also substantiates this, where high risk of diabetes was found among those having raised waist circumference. Studies have reported that despite having lean BMI, an adult Indian has more chances of having abdominal obesity. Also, the earlier studies in USA and UK have suggested that the insulin resistance in nonobese Asian Indians is due to the high percentage of visceral fat.[16,17]

When the occurrence of DM was analyzed with the levels of physical activity, it was found that those who were living sedentary life or involved in only mild activity were having 40% more risk than those involved in moderate and heavy activity, though the difference was statistically nonsignificant. It could be because of the fact that these subjects were diagnosed as diabetics before the present study and were on treatment. Thus, there are likely chances that they would have increased their physical activity as a part of their treatment.

Thus to summarize, the prevalence of self-reported diabetes in the present study was found to be 13.1%. It was found to be associated with increasing age, particularly after 45 years and the increasing waist circumference. The other factors such as sex, smoking habits, alcohol use, BMI ≥ 25 kg/m^2^ and sedentary lifestyle were found to be statistically nonsignificant factors.

The main limitation of this study was the use of very crude method for diagnosis of diabetes. This does not reflect the real prevalence, because on one hand, inclusion of subjects with impaired glucose tolerance who were on anti-diabetic treatment would have exaggerated the prevalence and on the other hand, the noninclusion of those on dietary control for diabetes would have underestimated it. However, this study gives the rough idea of the magnitude of the problem based on which a systematic study using standard diagnostic criteria can be undertaken. Meanwhile, the preventive measures like health education to promote regular physical activity, which reduces the prevalence of modifiable risk factors like obesity, particularly central obesity should be taken.
